# Application of *Lavandula angustifolia* Mill. Extracts for the Phytosynthesis of Silver Nanoparticles: Characterization and Biomedical Potential

**DOI:** 10.3390/plants13030333

**Published:** 2024-01-23

**Authors:** Ioana Raluca Șuică-Bunghez, Raluca Mădălina Senin, Ana Alexandra Sorescu, Mihaela Ganciarov, Iuliana Răut, Cristina Firincă, Mariana Constantin, Ioana Cătălina Gifu, Rusăndica Stoica, Irina Fierăscu, Radu Claudiu Fierăscu

**Affiliations:** 1 The National Institute for Research & Development in Chemistry and Petrochemistry—ICECHIM Bucharest, 202 Splaiul Independentei, 060021 Bucharest, Romania; raluca.bunghez@icechim.ro (I.R.Ș.-B.); ana-alexandra.sorescu@icechim.ro (A.A.S.); mihaela.ganciarov@icechim.ro (M.G.); raut.iuliana@icechim.ro (I.R.); cristina.firinca@icechim.ro (C.F.); mariana.constantin@icechim.ro (M.C.); catalina.gifu@icechim.ro (I.C.G.); rusandica.stoica@icechim.ro (R.S.); irina.fierascu@icechim.ro (I.F.); 2 Faculty of Biology, University of Bucharest, 91 Splaiul Independenței, 050104 Bucharest, Romania; 3 Faculty of Pharmacy, “Titu Maiorescu” University, 187 Calea Vacaresti, 040051 Bucharest, Romania; 4 Faculty of Horticulture, University of Agronomic Sciences and Veterinary Medicine of Bucharest, 59 Marasti Blvd., 011464 Bucharest, Romania; 5 Faculty of Chemical Engineering and Biotechnology, National University of Science and Technology Politehnica Bucharest, 1-7 Gheorghe Polizu St., 011061 Bucharest, Romania

**Keywords:** phytochemical analysis, lavender, UV-Vis, silver nanoparticles, SEM, XRD, DLS, antimicrobial

## Abstract

Nanotechnology can offer a series of new “green” and eco-friendly methods for developing different types of nanoparticles, among which the development of nanomaterials using plant extracts (phytosynthesis) represents one of the most promising areas of research. This present study details the use of lavender flowers (*Lavandula angustifolia* Mill., well-known for their use in homeopathic applications) for the biosynthesis of silver nanoparticles with enhanced antioxidant and antibacterial properties. Several qualitative and quantitative assays were carried out in order to offer an image of the extracts’ composition (the recorded total phenolics content varied between 21.0 to 40.9 mg GAE (gallic acid equivalents)/g dry weight (d.w.), while the total flavonoids content ranged between 3.57 and 16.8 mg CE (catechin equivalents)/g d.w.), alongside modern analytical methods (such as gas chromatography-mass spectrometry—GC-MS, quantifying 12 phytoconstituents present in the extracts). The formation of silver nanoparticles (AgNPs) using lavender extract was studied by UV-Vis spectroscopy, Fourier-transform infrared spectrometry (FTIR), scanning electron microscopy (SEM), X-ray diffraction (XRD), and dynamic light scattering (DLS)/zeta potential, with the selected nanoparticles having crystallite sizes of approx. 14.55 nm (AgNP-L2) and 4.61 nm, respectively (for AgNP-L4), and hydrodynamic diameters of 392.4 nm (for AgNP-L2) and 391.6 nm (for AgNP-L4), determined by DLS. A zeta potential of around −6.4 mV was displayed for both samples while presenting as large aggregates, in which nanoparticle clusters with dimensions of around 130–200 nm can be observed. The biomedical applications of the extracts and the corresponding phytosynthesized nanoparticles were evaluated using antioxidant and antimicrobial assays. The obtained results confirmed the phytosynthesis of the silver nanoparticles using *Lavandula angustifolia* Mill. extracts, as well as their antioxidant and antimicrobial potential.

## 1. Introduction

Green chemistry has become a sought-after alternative to conventional chemical routes due to its environmentally friendly methodologies and cost-effectiveness [1]. For the development of eco-friendly phytosynthesized metallic nanoparticles (NPs), numerous plants have been used; briefly, the method involves several important steps, all connected to the final morpho-structural characteristics of the NPs: collecting the plant material (the particular plant species, cultivars, growing and harvesting conditions influencing the composition of the extracts [2]), drying and processing of the vegetal material [3], obtaining the extracts (the extraction method of choice having a capital influence on the characteristic of the NPs [4]) and, finally, the mixing of the extract with a metallic salt (a step which introduces several others parameters connected to the NPs’ characteristics, such as the presence of light, mixing time and reaction temperature, metal salt concentration or the extract-to-metal-salt-solution ratio, among others [5]).

*Lavandula angustifolia* Mill. (commonly known as lavender) is a highly aromatic short shrub approximately 60 cm in dimension and originally native to the Mediterranean area, but in our days, it is being encountered all over the world, either as wild growing or as a cultivated ornamental or industrial plant (especially in lavender farms). Lavender plant parts, their extracts, or essential oils have been used since ancient times in several areas: in traditional or alternative medicine, in fragrances or pharmaceutical industries, or in aromatherapy due to their therapeutical properties, such as anticonvulsive, antiseptic, anti-inflammatory, antidepressant, antioxidant, and antimicrobial properties [6,7], or clinical benefits for the central nervous system [8]. The literature data [6,9,10] details several biomedical applications of lavender, including its use for the development of antibacterial, antifungal, antidepressive, or antitumoral agents.

The phytosynthesis of metal nanoparticles represents a subject of current interest in the scientific world due to their special properties (small sizes, large surface area and specific physical and chemical properties), making them suitable candidates for several areas with implications in our daily life [11,12].

Although the area of nanomaterial phytosynthesis represents a constantly developing research area, the number of papers dealing with nanomaterial phytosynthesis using lavender extracts is surprisingly low, considering that the plants are widespread. The literature data on the application of different lavender species presents the development of antibacterial silver nanoparticles using *L. stoechas* [13,14], cytotoxic silver nanoparticles using *L. dentata* [15] and *L. angustifolia* [16], as well as antioxidant nanoparticles using *L. angustifolia* [17]. Our group also previously presented a preliminary study on the phytosynthesis of antioxidant silver nanoparticles using *L. angustifolia* extract [18].

**Figure 1:**
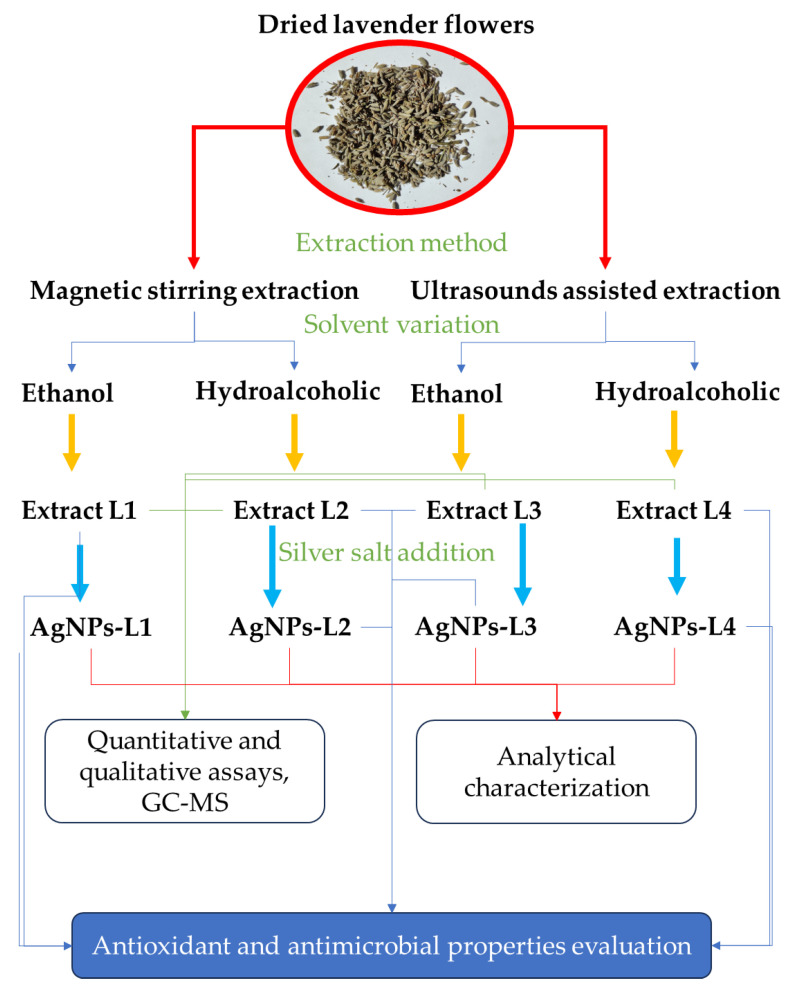
Schematic representation of the experimental procedure.

This present study aims to capitalize on these literature data, as well as on the known antimicrobial properties of lavender plants [19], describing a procedure (schematically presented in Figure 1) for the obtaining of natural extracts, their analysis, the phytosynthesis of silver nanoparticles and their analytical characterization, as well as the evaluation of the NPs in terms of their antioxidant and antimicrobial properties (against *Staphylococcus aureus*, *Escherichia coli*, and *Candida albicans*).

## 2. Results

### 2.1. Qualitative and Quantitative Analyses of Lavender Extract

Qualitative analyses are based on the color change reaction of the extract samples upon the addition of different chemical reagents. The qualitative assays provide general information on the presence/absence of different groups of phytochemicals (such as saponins, alkaloids, tannins, flavonoids, proteins, and amino acids), all bioactive compounds commonly found in the lavender extracts. The methods were applied according to the literature data [20,21,22] and are briefly presented in Section 4.

The results of the qualitative assays are presented in Table 1.

#### 2.1.1. Total Polyphenols Content

The total polyphenols content (TPC) of the extracts was determined using the Folin–Ciocalteu method [5] from the regression equation of the calibration curve (y = 0.0055x + 0.0205, R^2^ = 0.9997), prepared between 20–100 mg/L. The results were expressed as milligrams of gallic acid equivalents (GAE) per gram of dry matter of plant material (mg GAE/g d.w.). The results revealed the presence of relatively high amounts of polyphenols in the lavender extracts.

#### 2.1.2. Total Flavonoids Content

**Figure 2:**
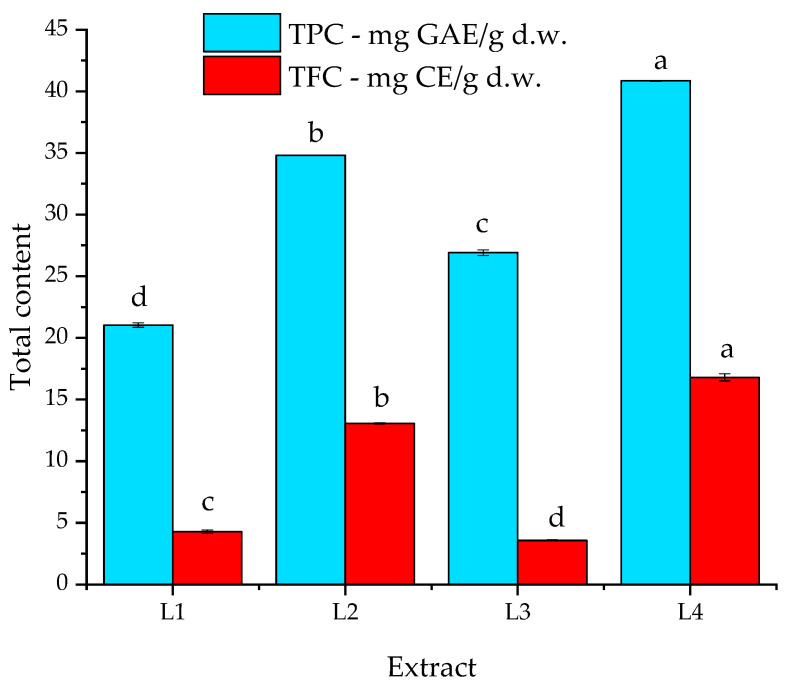
Graphical representation of total polyphenols content (TPC, mg GAE/g d.w) and total flavonoids content (TFC, mg CE/g d.w); values in the graph represent means ± SEM, *n* = 3 per treatment group; means in a series (TPC, respectively, TFC) without a common superscript letter differ (*p* < 0.05) as analyzed by one-way ANOVA and the Tukey post hoc test.

The total flavonoids content (TFC) of the samples was determined using a commonly applied method [5] from the regression equation of the calibration curve (y = 0.0033x − 0.0012; R^2^ = 0.9997) prepared between 20–100 mg/L. The results were expressed as mg catechin equivalents per gram of dry matter of plant material (mg CE/g d.w.), as presented in Figure 2.

As visible in Figure 2, the total polyphenols content (TPC) varied from 21.0 to approx. 40.9 mg GAE/g d.w., while the total flavonoids content (TFC) ranged between 3.57 and 16.8 mg CE/g d.w. for the analyzed extracts.

#### 2.1.3. GC-MS Results

For the detection and quantitative determination of the extracted phytoconstituents, was used a GC-MS chromatograph (Perkin Elmer–Clarus 500, PerkinElmer Inc., Shelton, CT, USA) with an Elite-5MS (5% diphenyl methyl polysiloxane stationary phase) column. The optimal GC-MS separation parameters were established, and the results were compared using the NIST Mass Spectral Library and literature data [23,24]. The GC-MS results are presented in Table 2.

### 2.2. Synthesis and Characterization of Silver Nanoparticles

**Figure 3:**
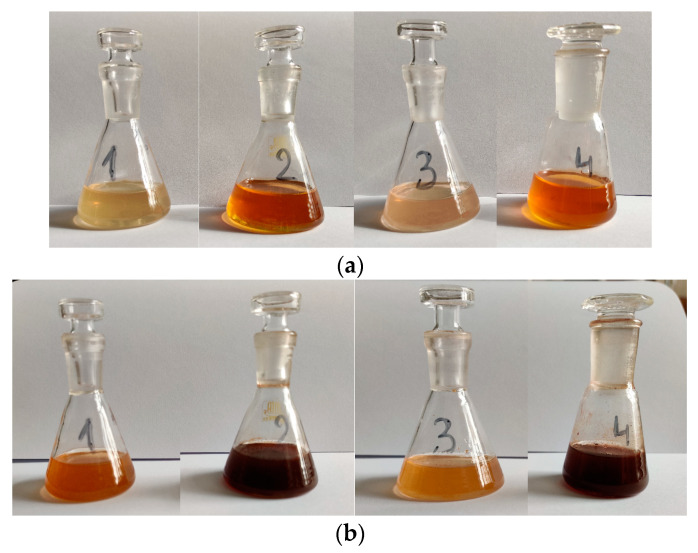
(**a**) Lavender extracts 1 to 4 (extracts L1 to L4, as described in Section 4); (**b**) phytosynthesized NP solutions 2 h after the addition of the silver salt.

The reduction of silver ions using the lavender extracts can be visually observed. The color of the extracts (Figure 3a) changed after the addition of AgNO_3_ (Figure 3b), from pale yellow to dark brown due to the excitation of surface plasmon vibrations in the metal nanoparticles.

**Figure 4:**
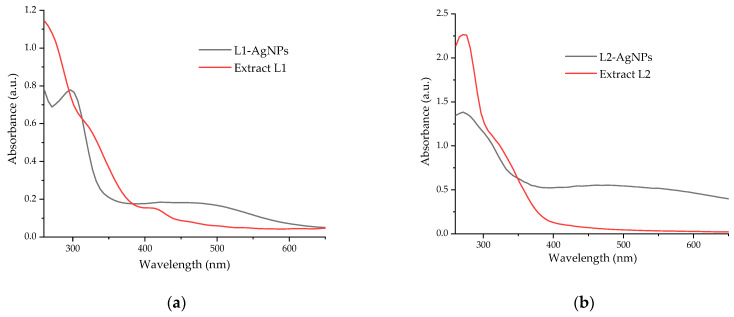

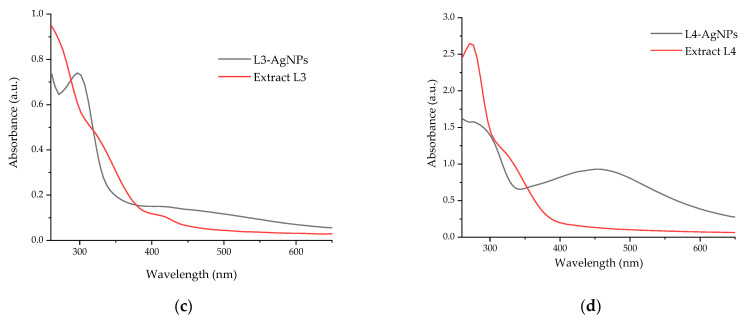
UV-Vis spectra of AgNP phytosynthesized, compared with the corresponding extracts: (**a**) L1, (**b**) L2, (**c**) L3, and (**d**) L4.

By the application of UV-Vis spectrometry, the reduction of silver metallic ions to silver nanoparticles can be monitored by the evaluation of specific peaks apparent in the region of 400–500 nm. Figure 4 presents the UV-Vis spectra obtained for the lavender extract, identifying the presence of the peaks specific to flavonoids and other phenolic compounds (between 280–330 nm), as well as the reduction of silver ions in the AgNP-extract solutions, confirmed by the presence of specific peaks in the region of interest [25,26].

**Figure 5:**
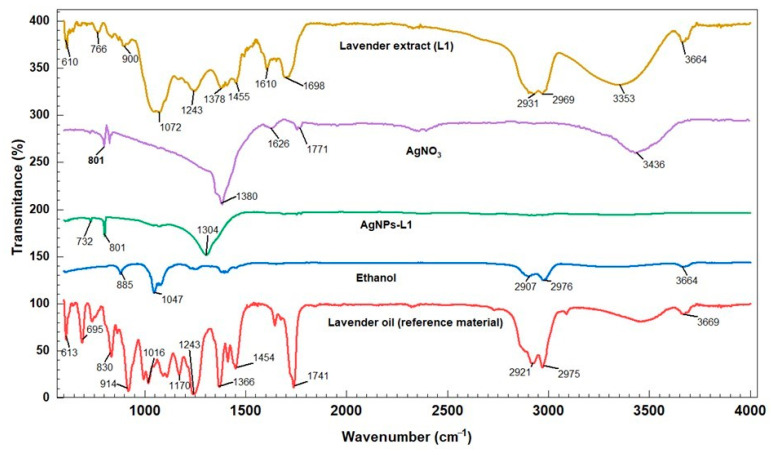
Comparison of FTIR spectra of extract L1 and its corresponding NP solution (AgNPs-L1) with the lavender oil, ethanol, and silver salt.

Fourier-transform infrared spectroscopy–attenuated total reflectance (FTIR–ATR) analysis was performed, having, as the main goal, the identification of the main groups of secondary metabolites that can be found in the extracts that could have a role in the phytosynthesis process. For exemplification, Figure 5 presents a comparison between extract L1, the developed NPs (AgNPs-L1), and a reference material (lavender oil). Also, the FTIR spectra of the solvent used (ethanol) and of the silver salt are presented for comparison purposes.

**Figure 6:**
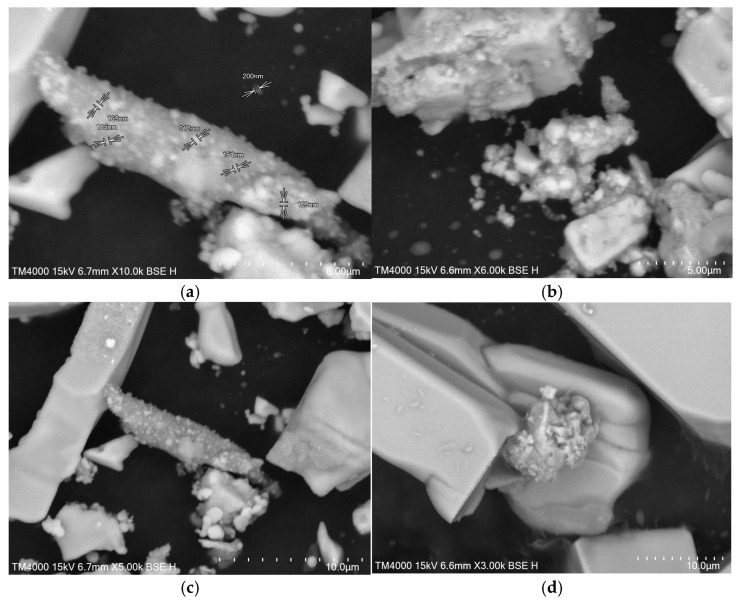
SEM micrograph of AgNP obtained using lavender extracts: (**a**) AgNP-L1; (**b**) AgNP-L2; (**c**) AgNP-L3; and (**d**) AgNP-L4.

In order to perform scanning electron microscopy (SEM) analyses, the solutions were ultrasonicated for 30 min and then were left to evaporate overnight at room temperature. The SEM images presented in Figure 6 show a high density of silver nanoparticles phytosynthesized using *L. angustifolia* flower extracts. The development of silver nanostructures is confirmed; however, considering the limitations of the technique, the NPs can be observed as large aggregates, in which clusters with dimensions of around 130–200 nm can be identified.

Considering the results of the UV-Vis evaluation of the NPs’ formation, as well as the registered total phenolics and total flavonoid contents, samples AgNP-L2 and AgNP-L4 were selected for further analytical studies (using dynamic light scattering—DLS and X-ray diffraction—XRD analyses).

**Figure 7:**
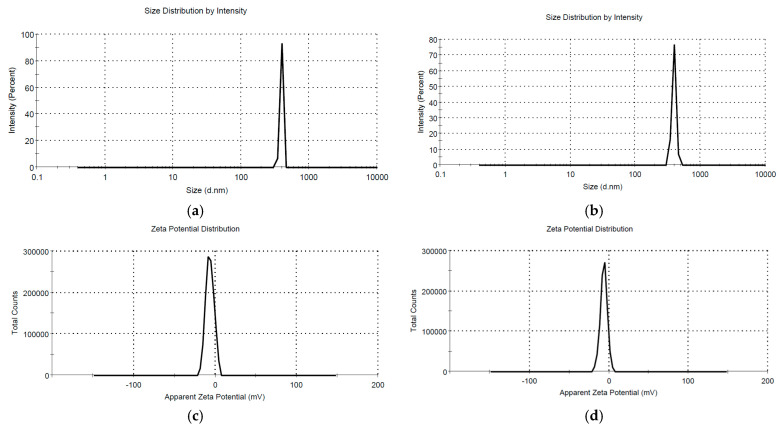
DLS analysis of AgNP obtained using lavender extracts: (**a**) AgNP-L2 and (**b**) AgNP-L4; and the zeta potential measurements of the samples: (**c**) AgNP-L2 and (**d**) AgNP-L4.

The DLS analysis revealed an average hydrodynamic diameter of 392.4 nm and a PdI of 0.614 for sample AgNP-L2, respectively, and an average hydrodynamic diameter of 391.6 nm and a PdI of 0.536 for sample AgNP-L4. The measured zeta potential of the two samples was −6.38 mV for sample AgNP-L2 and −6.40 mV for sample AgNP-L4, respectively (Figure 7c,d).

**Figure 8:**
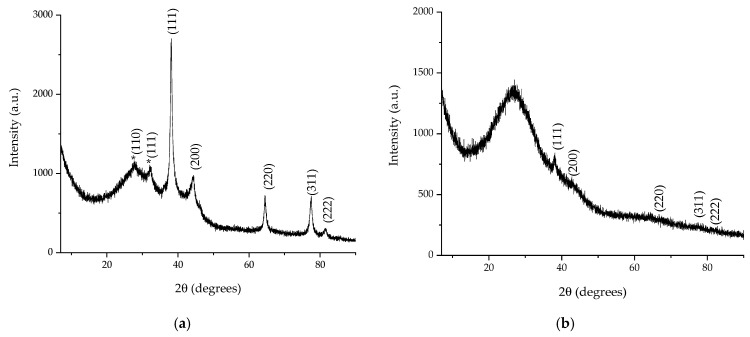
XRD diffractograms of AgNP obtained using lavender extracts: (**a**) AgNP-L2 and (**b**) AgNP-L4; the asterisk marks the maxima corresponding to the Ag_2_O phase.

The crystallographic analysis was performed following two aspects: first, to confirm the synthesis of silver nanoparticles, and, on the other hand, to evaluate the crystallite size (using the Debye–Scherrer equation). X-ray diffraction data for the two analyzed samples displayed five diffraction maxima corresponding to the diffraction planes: (111)—approx. 38.1°, (200)—approx. 44°, (220)—approx. 64°, (311)—approx. 77°, and (222)—approx. 81°, respectively, confirming the obtaining of Ag nanoparticles in a cubic crystalline system, identified based on ICDD entry 01-071-4613. A secondary phase appears for sample AgNP-L2 (marked with an asterisk in Figure 8), corresponding to a silver oxide state (Ag_2_O, ICDD card 01-078-5867), a phase most probably caused by the oxidation of the nanoparticles during the sample preparation for analysis.

The crystallite size, determined using Equation (2), revealed dimensions of 14.55 nm for sample AgNP-L2, respectively, and 4.61 nm for sample AgNP-L4.

### 2.3. Antioxidant and Antimicrobial Properties

**Figure 9:**
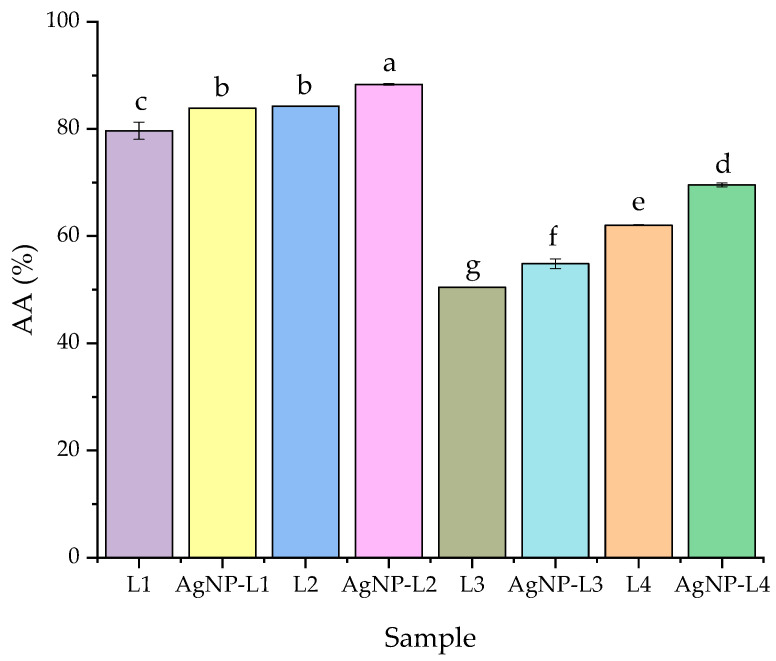
Results of the antioxidant properties evaluation: values are means ± SEM, *n* = 3 per treatment group. Means without a common superscript letter differ (*p* < 0.05) as analyzed by one-way ANOVA and the Tukey post hoc test.

Both the extract and nanoparticle dispersion samples were evaluated in terms of their antioxidant activity using the DPPH method [27], as presented in Section 4. The samples exhibited strong antioxidant properties, DPPH inhibition ranging between 50 and 84% for the plant extracts and between 54 and 88% for the silver nanoparticle solutions (Figure 9); the antioxidant properties were superior for the nanoparticle solutions, compared with the parent extracts. Also, the hydroalcoholic extracts and corresponding nanoparticles constantly presented superior results when compared with the alcoholic extracts and corresponding NPs.

**Figure 10:**
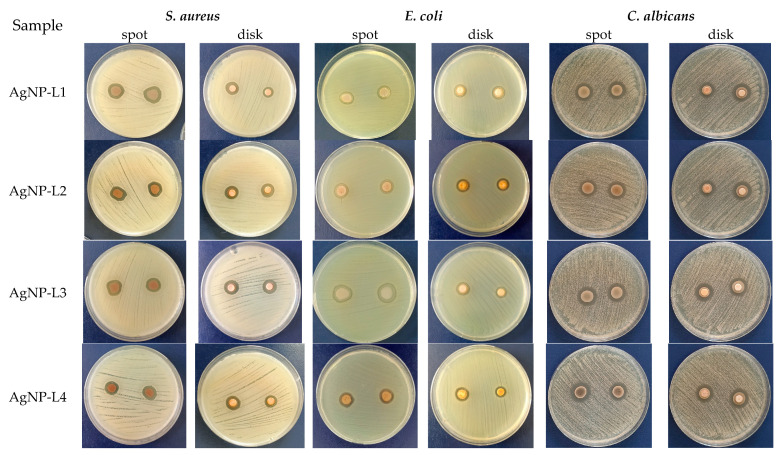
Antimicrobial activity of AgNP-lavender extract against *Staphylococcus aureus*, *Escherichia coli*, and *Candida albicans*.

The results of the antimicrobial assay (performed according to the protocol described in Section 4, using as-obtained samples) revealed that all the phytosynthesized NPs presented antimicrobial activity against the tested strains. Representative images of the Petri dishes used for the antimicrobial testing of the NPs are presented in Figure 10.

In the case of the AgNP-lavender extracts tested on *S. aureus and C. albicans*, the best efficiency was observed for AgNP-L1 (17.5 mm and 17 mm, respectively). The highest efficiency for the phytosynthesized nanoparticles was observed on the *C. albicans* strain, both by the spot and disk method. The results of the tests performed on *E. coli* showed that AgNP-L3 exhibited the best inhibition (17 mm) compared to AgNP-L1, AgNP-L2, and AgNP-L4. The diameters of the clear zone are presented in Table 3. For comparison, Table 3 also presents the results obtained for the extracts and the positive control (clindamycin 2 μg for *S. aureus*, ciprofloxacin 5 μg for *E. coli*, and clotrimazole 10 mg for *C. albicans*, respectively), as well as the solvents used for obtaining the extracts (pure ethanol—EtOH; hydroalcoholic solvent—H), and silver nitrate solution (at 10^−3^ M concentration).

## 3. Discussion

In the first step of this study, the lavender was extracted using different techniques (room temperature extraction under magnetic stirring for 24 h and ultrasound-assisted extraction at 40 °C for 2 h, respectively) and two different solvents (ethanol and a hydroalcoholic mixture, respectively). In the second step, the most laborious one, the lavender extracts were characterized through different analytical methods (FTIR, UV-Vis, and GC-MS), performed qualitative assays (for identifying the presence of carbohydrates, tannins, saponins, alkaloids, proteins, amino acids, and glycosides, respectively), and quantitative assays (for the determination of the total polyphenols and total flavonoid contents). The presence of phenolic compounds in the lavender extracts was determined by the Folin–Ciocalteu method, while the GC-MS analysis of the compounds, resulting from the extraction of the lavender samples, was performed in order to quantify the presence of different phytoconstituents. The concentrations of the 12 identified components were internally normalized, with response factors considered equal to unity. FTIR and UV-Vis analyses confirmed the reduction of Ag (I) to Ag (0) in the presence of the plant extract, the phytoconstituents acting as both reducing and capping agents.

FTIR–ATR was applied in order to identify the potential biomolecules involved in the Ag^+^ ions bioreduction and capping of the resulting NPs. In order to obtain a good signal/noise ratio, the FTIR transmission spectra of lavender extract before and after the bioreduction of Ag^+^ ions were recorded in the region of 600–4000 cm^−1^. In the lavender extract L1, lavender oil etalon, and AgNP-L1 samples, bands near 1698, 1455, and 1072 cm^−1^ were noted, which can be attributed to amides, proteins, and enzymes, respectively. All these compounds, together with other types (such as flavonoids or polyphenols), can synergistically contribute to the reduction of silver ions, according to the literature data [28,29]. The bands at 732 and 801 cm^−1^ in the FTIR spectra registered for AgNP-L1, respectively at 784 and 801 cm^−1^ in the FTIR spectra of AgNO_3_, are characteristics of silver.

The antioxidant capacity was measured using the DPPH radical scavenging method. All the analyzed lavender extracts showed high antioxidant capacity (with a recorded DPPH inhibition between approximately 50% and 84%). Upon phytosynthesis, all the NP extract solutions presented enhanced antioxidant activity (with an increase in the DPPH inhibition between 3.3% for samples AgNPs-L1/L1 and 12.43% for samples AgNPs-L4/L4). Overall, the recorded DPPH inhibition for the NP solutions ranged between 54.85% and 88.33%. The statistically significant enhancement of the antioxidant activity could be explained by the synergistic antioxidant activity of the phytoconstituents found in the extracts and of the obtained silver nanoparticles. Another important aspect is represented by the significant differences between the extraction method and the solvent used for extraction; it can be observed that the extraction method with a longer contact time leads to superior antioxidant activity (compared with the ultrasound-assisted extraction). The same conclusion is valid for the use of the hydroalcoholic solvent, compared with pure ethanol.

According to the literature data, the different phytochemicals found in plant extracts exhibit antioxidant properties, with some of the phytoconstituents having important roles as reducing or capping agents in the phytosynthesis process [30]. For example, Shahzadi et al. [30] recorded antioxidant activities between 25 and 42% DPPH inhibition for different concentrations of the *Tradescantia pallida* methanolic extract, which was enhanced in the case of phytosynthesized NPs (up to 77%).

The presented data are in good agreement with previously published results on the properties of other *Lavandula* species. Mahmoudi et al. [14] obtained 70 and 75% inhibitions for the 15 and 25 mg/mL *L. stoechas* methanolic extract, which was increased up to 85% for the NPs phytosynthesized using a 25 mg/mL extract. At the same time, the results presented in the current study are significantly higher compared with the ones presented by Kumar et al. [17], who obtained inhibitions of up to approximately 35% for both aqueous lavender leaf extract and the phytosinthesized AgNPs, with the results varying in a dose-dependent manner. 

The phytochemical assays were carried out in triplicate, and the results were calculated using specific calibration curves with very good regression indices. Our results are in agreement with those reported by Dobros et al. [31], who obtained a TPC between 16.73 and 28.01 for macerate extracts, the highest values being obtained for ultrasound-assisted extraction (UAE, between 23.11 to 32.82 mg GAE/g d.w.). The hydroalcoholic extracts showed higher total flavonoid contents, being comparable with the values reported by other authors of between 8.51 and 23.70 mg CE/g d.w for the UAE extracts [31].

The general conclusion of the assays is that the hydroalcoholic solvent is more efficient for the extraction of both phenolics and flavonoids (with statistically significant differences recorded by comparison with the use of ethanol as solvent), while as an extraction technique, the UAE proved to be more efficient for the extraction of both phenolics and flavonoids, compared with the magnetic stirring method (for the same solvent used). This is in apparent contradiction with the antioxidant assay results (regarding the efficiency of the extraction method); however, it must be considered that for the antioxidant activity, several other types of phytoconstituents also contribute besides the two classes quantified.

The next step of the experimental protocol is represented by the phytosynthesis and characterization of the obtained silver nanoparticles. This was achieved by modern analytic methods, including SEM, FTIR, UV-Vis spectrometry, DLS, and XRD, besides the evaluation of the antioxidant and antimicrobial activity enhancement of the NP solutions, compared with the parent extracts. The color of the AgNP-lavender extract solution became brown and presented an intense absorption band in the region that is specific to AgNPs. SEM analysis of the AgNP-lavender extract sample presented the formation of large aggregates nonuniform in shape, consistent with the hydrodynamic diameter determined by DLS.

The presence of much more well-defined UV-Vis spectra for samples AgNP-L2 and AgNP-L4, by comparison with AgNP-L1 and, respectively, AgNP-L3, led to the further characterization of the NPs phytosynthesized using the hydroalcoholic extracts. The selection was based on both the shape of the spectra and on the hypsochromic shift of the peaks corresponding to the AgNPs in the hydroalcoholic extracts, compared with the alcoholic extracts-mediated AgNPs. The characteristic peaks of silver nanoparticles suffered a hypsochromic shift (when comparing the nanoparticles phytosynthesized using similar conditions and different solvents: L2 vs. L1, respectively, and L4 vs. L3). Thus, for sample AgNP-L1, the characteristic peak was apparent at approximately 470 nm (with a weak secondary peak appearing around 415 nm); for sample AgNP-L2, it was at 463 nm; for sample AgNP-L3, it was at 457 nm; while for sample AgNP-L4 (the best-defined peak), it was centered at 454 nm. According to the literature data, the position of the characteristic peak can be correlated with the dimensions of the nanoparticles. In our case, this would suggest dimensions of over 72 nm for AgNP-L1, 68 nm for AgNP-L2, 64 nm for sample AgNP-L3, and 62 nm for sample AgNP-L4.

The lower dimensions can also be correlated with higher polyphenolics and flavonoid contents, as demonstrated by the phytochemical assays. The superior antimicrobial effect recorded for the NP samples obtained using the alcoholic extract is due, to a much greater extent, to the use of concentrated ethanol (a widely used antimicrobial agent), which counteracts the influence of lower-dimension NPs obtained in the hydroalcoholic extract.

From the presented antioxidant assay data, it can be observed that superior DPPH inhibition is obtained for the NPs phytosynthesized using hydroalcoholic extracts, compared with the ones developed using alcoholic extracts.

The results obtained are in line with those presented by Lin et al. [13] in their investigation of the antimicrobial activity of the AgNPs phytosynthesized using *L. stoechas* extracts, who reported that *C. albicans* was the most sensitive microbial species among the tested strains, followed by the Gram-positive bacterium *S. aureus*.

Regarding the nanoparticle characteristics, the results obtained in the present study suggest slightly higher dimensions of the particles, compared with the study of Belova et al. [16], as suggested by the position of the UV-Vis peak (under 450 nm in the cited study, compared with 454–470 nm in the current work). The cited authors also performed TEM analysis, obtaining diameters of around 35 nm.

Similar results were obtained by Kumar et al. [17], both in terms of the UV-Vis peak position and DLS results (although, in our case, the results suggest polydisperse solutions), who evaluated nanoparticles phytosynthesized using aqueous lavender extract. However, the results of the antioxidant assay are superior in the present work, which may be associated with the extraction method applied, which leads to the development of extracts with superior antioxidant potential.

The antioxidant and total phenolic results obtained are also similar to those obtained by Salayova et al. [29] regarding the plant extracts (especially for sample L4), although those assays are strongly influenced by the value of the cultivar, harvesting time, and season, as well as by a series of other environmental and extraction factors [32]. Similar results were obtained in terms of the hydrodynamic diameters (by DLS analysis) by Al Sufyani et al. [15] for the NPs obtained using *L. dentata* extract. The zeta potential recorded for the two analyzed samples revealed a relatively stable solution, the values recorded being in agreement with those obtained by Singh and Mijakovic [33] (−6.12 mV for the *Pseudomonas putida* KT2440-synthesized AgNPs), Jakinala et al. [34] (−7.12 mV for the NPs developed using *Mang mao* wings extract), and Alsareii et al. [35] (−6 mV for the NPs obtained using *Rhizophora apiculate* extract); the negative charge being attributed to the phytoconstituents acting as capping agents [34], contributing to the good colloidal nature and stability of the dispersion [35]. Theoretically, particles with values of a zeta potential over +30 mV or less than −30 mV are considered stable; however, when the determinations are carried out directly on the NP solutions, without any purification, values under those limits are commonly encountered [33,34,35].

The crystallite size obtained in the present work also supports the conclusion suggested by the phytochemical assays, respectively, that the ultrasound-assisted temperature extraction leads to extracts with higher contents in the secondary metabolites with a role in the phytosynthesis process, which in turn leads to smaller dimension nanoparticles, as there are significant differences between the crystallite size of the analyzed samples.

## 4. Materials and Methods

### 4.1. Materials

All the reagents used are commercially available: silver nitrate (used as a 10^−3^ M solution), ethanol (Merck KGaA, Darmstadt, Germany), DPPH (2, 2-diphenyl-1-picryl-hydrazyl-hydrate stable free radical), catechin, gallic acid (Sigma-Aldrich Chemie GmbH, Schnelldorf, Germany), and the Folin–Ciocalteu reagent (CPA Chem, Bogomilovo, Bulgaria). The reagents used for the qualitative assays are as follows: ferric chloride, sodium hydroxide, copper sulphate, acid acetic glacial, chloroform, NaOH, Hager, Benedict, Wagner and Millon, and sulfuric and chlorohydric acid (Merck KGaA, Darmstadt, Germany). Ultrapure water was obtained in our laboratory using a water purification system from Milli-QR Integral 30/10/15 Systems. The *Lavandula angustifolia* Mill. flowers were purchased from a local market.

### 4.2. Extraction Methods and Phytosynthesis Procedure

*L. angustifolia* flowers were dried to a constant mass, after which they were separated into 4 different samples, as presented in Table 4. For each sample, 1 g of dried flower material was weighed and transferred into a 50 mL Erlenmeyer flask, which contained ethanol (95%) or a hydroalcoholic mixture (ultrapure water: ethanol, 1:1) in order to release the intracellular material into the solution. The obtained lavender extracts were left to cool down at room temperature and filtered through a FILTRAK-grade 390 filter paper to obtain clear extracts.

For studying the extracts’ potential to phytosynthesize silver nanoparticles, the previously prepared extracts were mixed with the silver nitrate solution. An aqueous solution of silver nitrate (10^−3^ M AgNO_3_) was prepared and used for the phytosynthesis; 5 mL of lavender extract (as presented in Table 4) was mixed with 5 mL of AgNO_3_ aqueous solution, leading to a final silver concentration in each sample of 0.5 mM. The nanoparticle solutions were encoded from AgNP-L1 to AgNP-L4 (by reference to the corresponding extract).

### 4.3. Characterization Methods 

#### 4.3.1. Phytochemical Assays

A series of qualitative and quantitative assays were performed in order to confirm the presence of different types of compounds in the analyzed samples. The assays are briefly presented in Table 5 and Table 6.

#### 4.3.2. Analytical Methods

ATR-FTIR spectroscopy: For Fourier-transform IR spectroscopy, the spectra were collected using a Perkin Elmer Spectrum GX instrument (PerkinElmer Inc., Shelton, CT, USA). Spectra were registered using attenuated total reflectance (ATR) diamond crystal in the range of 4000–600 cm^−1^ at a spectral resolution of 4 cm^−1^.

UV-Vis spectroscopy analysis: The absorption spectra of the plant extracts and the silver nanoparticles were obtained with a double beam UV-Vis spectrophotometer Cintra 202 (GBC Scientific Equipment Ltd., Dandenong, VIC, Australia), in the wavelength range of 250–650 nm.

GC-MS: For the detection and quantitative determination of lavender compounds extracted (e.g., eucalyptol, linalool, camphor, terpinenol, linalylacetat, etc.), was utilized a GC-MS chromatography equipment (Perkin Elmer–Clarus 500) with an Elite-5MS (5% diphenyl methyl polysiloxane stationary phase) column. The 12 identified component concentrations were internally normalized with response factors, which were considered equal to unity.

The results were compared using the NIST Mass Spectral Library.

SEM technique: The morphology of the samples was studied using a TM4000plus II scanning electron microscope, SEM (Hitachi Ltd., Tokyo, Japan). For visualization, samples were deposited onto a carbon band and dried at room temperature. The electron accelerating voltage was 15 kV, and a standard vacuum was used in the sample chamber as specified by the manufacturer.

DLS: The hydrodynamic diameter and zeta potential of the silver nanoparticles were measured using the dynamic light scattering technique (Zetasizer Nano ZS, Malvern Instruments Ltd., Worcestershire, UK) at a scattering angle of 90° and 25 °C temperature, using intensity distribution.

XRD: The crystallographic analysis of the developed nanoparticles was performed using a 9 kW Rigaku SmartLab diffractometer (Rigaku Corp., Tokyo, Japan, with the operating parameters of 45 kV and 200 mA; CuKα radiation, 1.54059 Å), in scanning mode 2*θ*/*θ*, between 7° and 90° (2*θ*). Components were identified by comparison with ICDD data. The crystallite size was determined using the Debye–Scherrer equation:

(1) $$D_{P}=\frac{K\times \lambda }{\beta \times cos\theta }$$

where *Dp* represents the average size of the crystallites, *K* is the Scherrer constant (for cubic structures, *K* = 0.94), *β* is the width at a half-height of the diffraction maximum, *θ* is the Bragg angle, and *λ* is the wavelength (1.54059 Å in our case).

### 4.4. Evaluation of Biomedical Potential

For the determination of antioxidant activity, the DPPH assay was applied. A total of 0.5 mL of lavender extract sample or as-obtained silver nanoparticle solutions were mixed with 1 mL of 0.02 mg/mL DPPH solution. Afterwards, the mixtures were tested through absorbance readings at 517 nm. As a blank, we prepared 0.5 mL of bidistilled water with 1 mL of 0.02 mg/mL DPPH solution, which was read at the same wavelength.

The antioxidant activity (*AA* %) percentage was calculated using the formula:

(2) $$AA\left(\%\right)=\frac{AC-AS}{AC}\times 100$$

where *A_Control_* is the absorbance of the DPPH solution without a sample, and *A_Sample_* is the absorbance of the sample mixed with 0.02 mg/mL of the DPPH solution.

The antimicrobial activity was evaluated against pathogenic bacterial strains of Gram-positive *Staphylococcus aureus* (ATCC 25923), Gram-negative *Escherichia coli* (ATCC 25922), as well as yeast *Candida albicans* (ATCC 10231). All microbial strains used in the experiments were purchased from the German Collection of Microorganisms and Cell Cultures (DSMZ) (Braunschweig, Germany). The strains were selected, on the one hand, due to their pathogenicity and, on the other hand, due to their widespread use in the scientific literature as strains of choice in antimicrobial assays, which would allow future comparison of the results obtained [5,32,46].

The evaluation of the antimicrobial properties of lavender extracts and phytosynthesized AgNP was performed using the agar disk diffusion method through spot and disk variants [47,48,49,50]. The tests were performed in four replicates. Also, antibiotics and antifungals were used for the experiments, as well as the solvents used (ethanol and hydroalcoholic mixture) and the AgNO_3_ solution. As inoculum, a suspension in the sterile physiological water made from the microbial culture, with a turbidity of 1 × 10^8^ CFU/mL, adjusted nephelometrically (McFarland standard 0.5 = 1.5 × 10^8^ CFU/mL) was used. The bacterial strains were inoculated into a canvas with sterile swabs on solid Muller–Hinton agar (MHA), while *C. albicans* was inoculated on the Sabouraud dextrose agar medium (SDA).

Aliquots (20 µL) of the undiluted samples were placed in the Petri dishes inoculated with the microbial strains. The plates were incubated for 24 h at 37 °C in the case of bacterial strains and at 28 °C in the case of yeast. Antimicrobial activity was evaluated by measuring the zones of inhibition (the halo around the spot/disk) after 24 h.

The composition of MHA (g/L) was: 2, meat extract; 1.5, starch; 17.5 casein hydrolysate; 17, agar; and 1000 mL of distilled water (final pH = 7.2–7.4). The composition of SDA medium (g/L) was: 40, dextrose; 10, peptone; 15, agar; and 1000 mL of distilled water (the final pH = 5.6 ± 0.2).

### 4.5. Statistical Analysis and Data Representation

The determinations were carried out through multiple parallel determinations (as detailed for each method); the obtained data were analyzed for their statistical significance using analysis of variance (one-way ANOVA) and Tukey’s post hoc test to determine significant differences between the means. Significant differences were set at *p* ≤ 0.05. The results presented in this manuscript represent a means ± standard error of the mean (SE) of multiple independent determinations.

The obtained data were represented using a dedicated graphical representation software, OriginPro 2018 Data Analysis and Graphing Software (version 95E, OriginLab Corporation, Northampton, MA, USA).

## 5. Conclusions

This current study presents the differences recorded in terms of the composition and phytosynthesis potential for the lavender extracts obtained by varying the extraction method (magnetic stirring at 20 °C and ultrasound-assisted temperature extraction) and the solvent used (ethanol and the hydroalcoholic mixture, respectively).

In terms of the phytoconstituents, the ultrasound-assisted temperature extraction and the use of the hydroalcoholic mixture as a solvent led to much higher levels of total phenolics (up to 40.9 mg GAE/g d.w.) and total flavonoids (up to 16.8 mg CE/g d.w.). The results were partially correlated with the results of the antioxidant activity evaluation (the hydroalcoholic extracts presented superior results compared with the alcoholic extracts). Overall, the analyzed extracts and nanoparticle solutions showed high antioxidant capacity (with a recorded DPPH inhibition ranging between approximately 50% and 84% for the extracts and between approximately 54% and 88.3% for the nanoparticle solutions). The phytosynthesis process led to an enhancement of the antioxidant activity of the corresponding extract (up to a 12.43% increase of the DPPH inhibition being registered); the statistically significant enhancement of the antioxidant activity was explained by the synergistic antioxidant activity of the phytoconstituents found in the extracts and of the obtained silver nanoparticles.

Regarding the analytical characterization of the nanoparticle solutions, the best-defined UV-Vis characteristic peak was obtained for the NPs phytosynthesized using UAE extraction and hydroalcoholic mixture as the solvent.

Selected nanoparticle samples (obtained using the hydroalcoholic solvent) were also characterized using XRD and the DLS/zeta potential. The results support the conclusion that the extracts obtained by the ultrasound-assisted temperature extraction lead to smaller dimensions compared with the magnetic stirring-assisted room temperature extraction.

The lower dimensions of the nanoparticles can also be correlated with higher polyphenolics and flavonoid contents. The developed nanoparticle solutions had superior antimicrobial efficiency against all tested microbes compared with the corresponding extracts.

## Figures and Tables

**Table 1:** Qualitative screening of the extracts (where: + = presence; − = absence); the encoding of the extracts (L1–L4) is presented in Section 4.

Class of Compounds	Assay	Extract L1	Extract L2	Extract L3	Extract L4
Carbohydrates	Carbohydrates (general)—Molisch	Purple-red solution (+)	Purple solution (+)	Dark purple solution (+)	Purple solution (+)
	Carbohydrates (reducing sugars)—Benedict	Brick-red precipitate (+)	Dark red precipitate (+)	Dark red precipitate (+)	Red precipitate (+)
	Carbohydrates (reducing sugars)—Fehling	Brown-yellow solution (+)	Brown-yellow solution (+)	Brown-yellow solution (+)	Brown solution (+)
	Carbohydrates (reducing sugars)—Trommer	Red precipitate (+)	Red precipitate (+)	Red precipitate (+)	Red precipitate (+)
	Carbohydrates (reducing sugars)—Tollens	Black precipitate (+)	Black precipitate (+)	Black precipitate (+)	Black precipitate (+)
	Carbohydrates (reducing sugars)—Moore	Red-brown solution (+)	Red-brown solution (+)	Red-brown solution (+)	Red-brown solution (+)
	Carbohydrates (hexose sugars)—Seliwanoff	Cognac-red solution (+)	Red solution (+)	Dark red solution (+)	Dark red solution (+)
	Carbohydrates (hexose sugars)—Cobalt chloride	Lower layer-blue precipitate, upper layer-pink solution (+)	Lower layer-blue precipitate, upper layer-pink solution (+)	Lower layer-blue precipitate, upper layer-light pink solution (+)	Lower layer-blue precipitate, upper layer-light pink solution (+)
	Carbohydrates (hexose sugars)—Ammonium molibdate	Blue-green solution (+)	Blue-green solution (+)	Blue-green solution (+)	Blue-green solution (+)
Tannins		Dark green solution (−)	Green solution (−)	Light green solution (−)	Light green solution (−)
Saponins		0.6 cm foam layer (+)	0.8 cm foam layer (+)	0.6 cm foam layer (+)	1.2 cm foam layer (+)
Alkaloids	Alkaloids—Wagner (Red-brown solution)	(+)	(+)	(+)	(+)
	Alkaloids—Mayer (Light yellow solution)	(+)	(+)	(+)	(+)
	Alkaloids—Hager (Clear yellow solution)	(+)	(+)	(+)	(+)
Proteins and aminoacids	Aminoacids—Ninhydrin test (Opalescent white-yellow solution)	(+)	(+)	(+)	(+)
	Aminoacids—test for cysteine (Red-brown solution, black precipitate)	(+)	(+)	(+)	(+)
	Proteins—biuret test (Green solution)	(+)	(+)	(+)	(+)
	Proteins—Xantoprotein test (Light brown solution)	(+)	(+)	(+)	(+)
Glycosides	Glycosides (cardiac)—FeCl_3_ reagent (Orange-yellow solution)	(−)	(−)	(−)	(−)
	Glycosides (cardiac)—Keller–Killani test (Brown ring at the interface)	(−)	(−)	(−)	(−)
	Glycosides (anthraquinonic)—Borntrager test (Colorless lower layer, opalescent white upper layer)	(−)	(−)	(−)	(−)

**Table 2:** The compounds identified and determined by GC-MS. Encoding of the extracts (L1-L4) is detailed in Section 4.

No.	Retention Time tR (min)	Identified Compounds	Extract Compounds Conc. %			
			Extract L1	Extract L2	Extract L3	Extract L4
1	9.66	Eucalyptol	1.99	5.41	7.33	7.25
2	10.31	Linalool	14.3	51.8	45.1	51.6
3	11.01	2-Bornanone	2.85	9.22	9.73	9.45
4	11.23	Butanoic acid, hexyl ester	0.21	0.55	0.79	0.73
5	11.27	Endo-borneol	1.19	3.81	4.01	3.95
6	11.33	Terpinen-4-ol	1.31	3.40	4.30	3.77
7	11.55	Hexanedioic acid, bis(2-ethylhexyl) esters	4.20	0.49	0.57	0.77
8	11.81	Linalyl acetate	4.99	14.2	15.1	13.5
9	13.69	Caryophyllene	0.97	1.36	2.62	1.25
10	14.99	Hexanedioic acid, dioctyl esters	65.9	7.50	8.30	5.61
11	15.70	Alpha-bisabolol	0.84	1.27	1.15	1.29
12	17.85	Dibutyl phthalate	1.21	1.02	0.98	0.82

**Table 3:** The diameters of the AgNP samples.

Microbial Strain	AgNP-L1		L1		AgNP-L2		L2		AgNP-L3		L3		AgNP-L4		L4		AgNO_3_	EtOH	H	+ Control
	Spot	Disk	Spot	Disk	Spot	Disk	Spot	Disk	Spot	Disk	Spot	Disk	Spot	Disk	Spot	Disk				
	(mm)		(mm)		(mm)		(mm)		(mm)		(mm)		(mm)		(mm)		(mm)	(mm)	(mm)	(mm)
*Staphylococcus aureus*	17.5	13	0 *	0 *	15	15	0 *	0 *	15	15	0 *	0 *	14	13	0 *	0 *	14	ND **	ND **	25
*Escherichia coli*	13.5	13.5	12	20	14	14	0 *	0 *	17	12.8	16	16	15	13.5	0 *	0 *	12	19.5	0	31
*Candida albicans*	17	16	16.5	19	16	16	0 *	0 *	16.5	16	13	17	16	15.5	0 *	0 *	19	19.5	9	17.5

*—Extracts showing no antimicrobial efficiency (no inhibition zone); as such, the results are presented as 0. **—experiments were not performed for the solvents associated with the ethanol and hydroalcoholic mixture extracts, due to the fact that the corresponding extracts showed no inhibition zone; ND—not determined.

**Table 4:** Synthesis of lavender extracts.

Encoding	Extraction Method	Extraction Solvent	Temperature (°C)	Time (h)
L1	Magnetic stirring (M)	EtOH (E)	20	24
L2	Magnetic stirring (M)	Hydroalcoholic mixture (H) (1:1, EtOH: ultrapure water)	20	24
L3	Ultrasound bath (U)	EtOH (E)	40	2
L4	Ultrasound bath (U)	Hydroalcoholic mixture (H) (1:1, EtOH: ultrapure water)	40	2

**Table 5:** The qualitative assays used in this study.

Assay	Procedure	Positive Test	Reference
Carbohydrates (general)—Molisch	1 mL of Molisch reagent (a solution of α-naphthol in ethanol) is added to 2 mL of aqueous extract; a few drops of concentrated sulfuric acid are slowly dripped, and the resulting solution is shaken carefully.	The appearance of a violet ring at the interface of the two liquids indicates the presence of carbohydrates in the aqueous extracts.	[36]
Carbohydrates (reducing sugars)—Benedict	To about 5 mL of the reagent in a test tube are added 8 drops of the sample; the sample is heated to boiling, kept at this temperature for 1–2 min, and allowed to cool.	The appearance of a precipitate occurs (red, yellow, or green); for low reducing sugar content, the precipitate forms only upon cooling.	[37]
Carbohydrates (reducing sugars)—Fehling	2 mL of the sample is added to 5 mL of Fehling’s solution in the test tube. Heat the tube in a water bath.	Appearance of a brown precipitate.	[38]
Carbohydrates (reducing sugars)—Trommer	To 1 mL of the sample, 1 mL of 2 M NaOH is added and mixed; CuSO_4_ solution is added drop-by-drop to keep the blue precipitate of Cu(OH)_2_ still soluble. When the precipitate appears, the CuSO_4_ addition is stopped, and the sample is heated in a boiling water bath.	Formation of an orange-red colored precipitate of cuprous oxide occurs.	[39]
Carbohydrates (reducing sugars)—Tollens	To 1 mL of 0.1 M AgNO_3_ solution is added dropwise 2M of water ammonia solution. After mixing, the initial AgOH precipitate will solubilize, forming [Ag(NH_3_)_2_]OH complex compound. To the solution, add 5 drops of the sample, mix, and heat in a boiling water bath.	The appearance of a silver mirror on the tube walls occurs.	[38]
Carbohydrates (reducing sugars)—Moore	To 2 mL of the sample is added an equal volume of 5% NaOH, and the mixture is boiled for 5 min.	The solution initially has a yellow coloration that changes to reddish-brown.	[40]
Carbohydrates (hexose sugars)—Seliwanoff	To 5 mL of Seliwanoff ’s reagent is added 1 mL of the sample; the mixture is heated using boiling water for 5 min.	A cherry-red color shows the presence of ketoses (fructose sugar). Ketopentoses yield a blue-green color, while aldoses and disaccharides give no shading change.	[38]
Carbohydrates (hexose sugars)—Cobalt chloride	3 mL samples are mixed with 2 mL of cobalt chloride, and the solution is boiled. After cooling, drops of 4% NaOH solution are added.	Greenish-blue solution (glucose), purplish-violet solution (fructose), or the upper layer turns greenish-blue, while the lower layer is purplish (both glucose and fructose).	[40]
Carbohydrates (hexose sugars)—Ammonium molibdate	To 2 mL of the sample, 2 mL of ammonium molybdate solution is added, and the solution is heated.	A bluish-green solution is formed.	[40]
Tannins	1 mL of the sample and 2 mL of 5% ferric chloride are mixed.	A dark blue or greenish-black color appears.	[41]
Saponins	2 mL of the sample and 2 mL of distilled water are shaken for 15 min in a graduated cylinder.	A 1 cm foam layer is a positive response to the presence of saponins.	[42]
Alkaloids (*Wagner test*)	1 mL of the sample and 1 mL of Wagner’s reagent (iodine in potassium iodide solution) react.	If a reddish-brown precipitate is formed, it indicates a positive reaction.	[43,44]
Alkaloids (*Mayer test)*	To 1 mL of the sample, 2 mL of concentrated HCl is added, followed by a few drops of Mayer’s reagent (a solution of mercuric chloride and potassium iodide in water).	A green color or white precipitate indicates the presence of alkaloids.	[40]
Alkaloids (*Hager test)*	2 mL of the sample and 2 mL of Hager’s reagent (a saturated aqueous solution of picric acid) are mixed together.	A yellow precipitate indicates a positive test.	[40]
Proteins (*Biuret test*)	3 mL of the sample, 3 mL of 4% sodium hydroxide solution, and a few drops of 1% copper sulfate are added to form a purple solution.	A purple solution indicates the presence of proteins.	[45]
Proteins (*Xanthoproteic test*)	3 mL of the sample and 1 mL of concentrated H_2_SO_4_ is slowly dropped.	If a white precipitate appears that turns yellow upon boiling and orange after 1 mL of NH_4_OH solution is added, this indicates protein presence.	[40]
Aminoacids (*Ninhydrin test*)	3 mL of the sample is mixed with three drops of 5% lead acetate solution and then heated.	A purple or blue coloration indicates a positive reaction.	[40]
Aminoacids (*cysteine test*)	5 mL of the sample is boiled with a small amount of 40% NaOH, and a few drops of 5% lead acetate solution are added.	A black precipitate is formed.	[39]
Glycosides (*FeCl_3_ test*)	1 mL of the sample, 1 mL of FeCl_3_ reagent (1 mL of 5% FeCl_3_ solution mixed with 99 mL of glacial acetic acid), and a few drops of concentrated H_2_SO_4_.	If it gives a greenish-blue color that appears in time, the sample presents glycosides.	[40,44]
Glycosides (*Keller–Killiani test*)	5 mL of the sample, 2 mL of glacial acetic acid + a drop of FeCl_3_ solution, and 1 mL of concentrated H_2_SO_4_.	If a brown ring appears and often a purple ring appears below, glycosides are present.	[40]
Glycosides (*Borntrager test*)	2 mL of the sample reacts upon boiling with 2 mL of H_2_SO_4_. The solution is filtered, and equal volumes of chloroform are added and shaken vigorously, and two layers can be clearly observed.	The organic layer is separated, and ammonia is added to form a pinkish-red color as a sign of a positive reaction.	[40]

**Table 6:** The procedure of quantitative methods.

Assay	Reagents	Monitoring and Calibration	Reference
Total flavonoids content/(TFC)	1 mL of extract + 4 mL of ultrapure water and 0.3 mL of NaNO_2_ (5% *w*/*v*). After 5 min, 0.3 mL AlCl_3_ (10% *w*/*v*). After another 5 min, 2 mL of 1M NaOH and 24 mL of ultrapure water. Then, 30 min of incubation at room temperature.	λ = 510 nm at UV-Vis; Catechin curve calibration standard	[45]
Total polyphenols content/(TPC)	1 mL of diluted extract and 5 mL of Folin–Ciocalteu reagent. After 8 min, 4 mL of Na_2_CO_3_. Then, 60 min of incubation at room temperature.	λ = 765 nm at UV-Vis; Gallic acid curve calibration standard	[46]

## Data Availability

The data presented in this study are available on request from the corresponding authors.
